# Assessment of Body Composition, Lower Limbs Power, and Anaerobic Power of Senior Soccer Players in Portugal: Differences According to the Competitive Level

**DOI:** 10.3390/ijerph18158069

**Published:** 2021-07-30

**Authors:** Diogo Tereso, Rui Paulo, João Petrica, Pedro Duarte-Mendes, José M. Gamonales, Sergio J. Ibáñez

**Affiliations:** 1 Department of Sport and Well-Being, Polytechnic Institute of Castelo Branco, 6000-084 Castelo Branco, Portugal; ruipaulo@ipcb.pt (R.P.); j.petrica@ipcb.pt (J.P.); pedromendes@ipcb.pt (P.D.-M.); 2 Sport, Health and Exercise Research Unit (SHERU), Polytechnic Institute of Castelo Branco, 6000-266 Castelo Branco, Portugal; 3 Research in Education and Community Intervention (RECI), 3515-776 Viseu, Portugal; 4 Research Group in Optimization of Training and Sport Performance (GOERD), Faculty of Sports Sciences, University of Extremadura, 10003 Caceres, Spain; josemartingamonales@gmail.com (J.M.G.); sibanez@unex.es (S.J.I.)

**Keywords:** body composition, lower limbs power, anaerobic power, soccer, competitive level

## Abstract

Background. During a soccer game, the most diversified stimuli occur all the time, the physical condition level plays a determinant role, and there may be variations according to the competitive level. In this sense, the present study aimed to verify differences in body composition, lower limbs power, and anaerobic power, comparing senior soccer players of different competitive levels. Methods. Participants were 81 players belonging to six soccer teams, aged between 18 and 35 years, with a mean age of 23.14 ± 4.23 years, who were divided into three distinct competitive levels: Elite, Sub-Elite and Non-Elite. The players performed bioimpedance evaluations on a tetrapolarInbody270 scale (body composition), the Countermovement Jump (CMJ) through the ChronoJump (lower limbs power), and Running Anaerobic Sprint Test (RAST) (anaerobic power). Results. Based on the competitive level analysis, we verified that the players present body composition values similar to each other regardless of the competitive level in which they play. Concerning the performance evaluations, we verified that the elite players present higher values of highest jump (*p* = 0.012; d = 0.76, moderate; and *p* = 0.022; d = 0.71, moderate) and maximum force produced (*p* = 0.05; d = 0.64, moderate; and *p* = 0.002; d = 1.00, moderate), together with higher values of anaerobic power (*p* < 0.001; d = 2.43, very large; and *p* < 0.001; d = 2.22, very large), compared to the others. Conclusions. We can thus conclude that there is a homogeneity regarding the body composition of soccer players, regardless of their competitive level; in turn, elite players show better performance indicators in all variables.

## 1. Introduction

Increasingly, soccer is a competitive sport that requires excellent relevance to physical capabilities, especially when talking about high level, we have as a reference the investigations that focus on monitoring players [1]. During a game, the most diverse stimuli occur all the time, the level of physical condition plays a decisive role, and there may be variations depending on the competitive level. Given the evolution of soccer, which is becoming more and more scientific, with the increase of physical, technical, and tactical demands, we try to know the actual behavior of specific variables and improve the training methods so that we can make a transfer closer to the specific situations of the game. However, training continues to be seen in a general way, and it is necessary to understand that the actions that occur during a game present totally different physical demands between competitive levels where the stimuli vary in frequency and complexity, being important to have a division of training between sectors and positions [2].

Nowadays, the evolution of technology applied to the world of soccer has become an important factor in the control of training and game, from programs to control distances covered, heart rate (HR) monitors, intensity, and fatigue level, all this allows better monitoring, evaluation, and control of the athletes’ evolution. Considering these aspects, it may be important to have a professional specialized in this type of work in any team. It is natural that with these different demands, the players themselves develop different characteristics. However, a similar base is fundamental. The body composition and the levels of aerobic capacity are completely different from sports that present, in their majority, standard motor movements, having a greater specificity and individuality in the physical preparation of the players for the effective accomplishment of specific actions [2,3]. Thus, when we talk about high performance, it is illusory to think about the evolution of sports performance and all the variables related to the players without understanding them as factors that condition the improvement of individual and collective performance [2,4].

Body composition is closely related to the players’ ability to achieve maximum performance in all their actions in the game, whereas rule teams with higher fitness levels and low-fat percentages play in the best leagues and championships [5,6]. In this way, the corporal composition is an indispensable factor for soccer players’ physical fitness. Several studies conclude the incompatibility between competitive excellence and high levels of subcutaneous adiposity since excess adipose tissue acts as undesirable weight in motor actions, in which the body mass must be continuously lifted against gravity and may substantially decrease the player’s performance [7].

Lower limbs power (vertical jump) varies from player to player. Carvalho [8] mentions that this explosive capacity is the key to many sports, especially those that require speed, agility, speed, and explosive force. On average, during a game, each player performs about 15 jumps in both defensive and offensive actions; the positions that usually perform a higher number of jumps are the Goalkeeper (GK) and Centre-back (CB). Thus, the stimuli provided by the game are not considered sufficient for this skill to be improved [9]. The maximum performance that a player can achieve in the execution of the vertical jump becomes fundamental for their success in the sport in which they are inserted, as is the case of soccer [10].

During a soccer game, elite players travel on average between 9 and 12 km [11,12]. However, due to its acyclic nature and the intense search for the ball, we can call it a high-intensity intermittent sport, in which there is a large volume of motor actions [13]. According to Ekblom [14], 8–18% of the total distance that a player covers per game is done at maximum intensity, suggesting that the anaerobic metabolism is essential for the performance during the game. The anaerobic capacity of an individual is characterized by the ability to regenerate ATP from other energy pathways than the oxidative pathways, being important to be able to perform, resist and repeat the actions of exercises of high intensity and short duration, predominantly alactic [15].

This study meets the lack of research in Portugal on the importance of these variables on individual and collective performance and on the professional ascension that a player may achieve in his sporting career. It is crucial to understand if the evaluated characteristics differ and are conditioned by the competitive level of each player. In a certain way, this study assumes scientific relevance by evaluating several components that interfere in sports performance, evaluating players of different competitive levels and with different physiological demands. Having as an objective, the understanding of the rise of a player to higher levels or their sports performance can be justified by these factors, or other factors that are not physical and trainable, trying, this way, to help to understand the main differences in physical performance between them.

This study aimed to evaluate the body composition, lower limbs power, and anaerobic power of senior soccer players in Portugal, intending to verify if the higher the competitive level of a player, the better his results in these variables. It was advanced as a hypothesis that players with higher competitive levels present better body composition values, best performance in the vertical jump (lower limbs power), and better values of anaerobic power.

## 2. Materials and Methods

### 2.1. Study Design

This research fits into a cross-sectional type of study. According to Rouquayrol [16], it is the most used type of research in this area and is based on the epidemiological study in which factors and effects are observed in the same historical moment.

According to Diehl and Tatim [17], the quantitative method was used based on quantification, both in data collection and in their treatment, using statistical techniques to avoid possible distortions in the analysis and interpretation of results, offering a more significant margin of safety.

### 2.2. Participants

Initially, 121 players accepted to participate in the study. After removing missing values, dropout, and missing cases (33.1%), data from 81 subjects were analyzed. Missing and dropout values were removed because some players did not fully complete the data collection protocol due to different situations. Some of them were dismissed during data collection, some were absent, and others were injured during the process or, according to the management of the players, where given the periodization established by each of the technical teams.

A total of 81 players participated in this study (*n* = 81), belonging to six soccer teams, aged between 18 and 35 years, with a mean age of 23.14 ± 4.23 years. The players were divided into three groups [18], according to their competitive level: Non-Elite (3 training sessions per week of 90 min)—30 federated players competing in the District Soccer Championship, with an average age of 22.57 ± 4.75 years; Sub-Elite (4 to 5 training sessions of 90 min per week)—30 federated players competing in the Portuguese Soccer League, with an average age of 21.93 ± 3.08 years; and Elite (5 to 7 training sessions of 90 min per week)—21 federated players competing in the 2nd Professional Soccer League, with an average age of 25.67 ± 3.95 years.

After selecting the teams and their respective players, we defined the following exclusion criteria: athletes under 18 years of age, and athletes who refused to participate. It should be noted that not all the players did all the evaluations due to the effort management, considering the periodization of the technical teams.

### 2.3. Instruments

To carry out the present study, we initially applied a questionnaire of anamnesis (name, field position, injury history, and competitive level, among others) to define each of the groups and characterize the sample. It should be noted that the data were collected by the same team of researchers, adequately trained, using the defined protocols.

#### 2.3.1. Body Composition

Body composition was assessed using an InBody 270 bio-impedance scale with an 8-Electrode Tetrapolar Electrode System with frequencies of 20 and 100 kHz, allowing the values of Skeletal Muscle Mass, Fat-free Mass, Fat Mass, Body Fat Percentage, and BMI to be obtained [19]. A portable stadiometer was used to enter the height value on the scales before going on the scales.

#### 2.3.2. Countermovement Jump (CMJ)

To obtain the performance values of each player, the ChronoJump platform was used along with the protocol proposed by Bosco, Luhtanen, and Komi [20] and Markovic et al. [21]. The lower limbs power values were collected using the vertical jumps of each player, in which it was possible to obtain the height reached and the maximum force produced in each jump by performing the Countermovement Jump (CMJ) method.

#### 2.3.3. Running Anaerobic Sprint Test (RAST)

**Figure 1:**
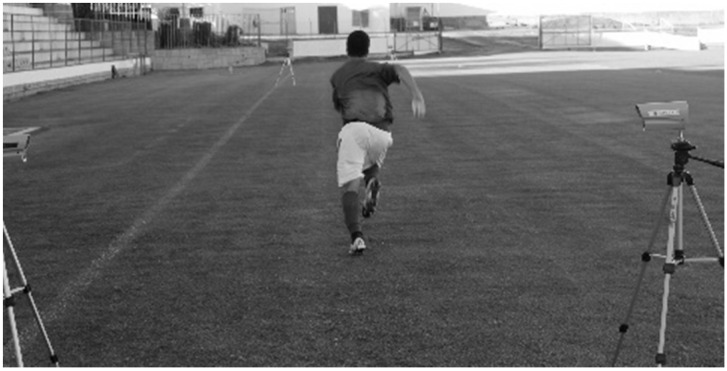
Evaluation of anaerobic power through the Running Anaerobic Sprint Test (RAST).

Regarding the tests that concern the anaerobic power of each player, the Running Anaerobic Sprint Test (RAST) was implemented [22,23]. The test consists of running six times the distance of 35 m in the shortest time possible, with a 10-s interval between each repetition; the output always starts in a static position (Figure 1). The time of each run was measured through the device Microgate Timing System—Race time 2. The anaerobic parameters determined were maximum power, minimum power, average power, and fatigue index.

### 2.4. Procedures

After the sports institutions’ approval and the scheduling of the assessments, the study was presented to the whole group, where the objectives and procedures were explained. All ethical procedures were taken into account, and the study was approved by the institution’s technical-scientific committee. Once the qualified players to participate in the tests were defined, an informed consent form and anamnesis sheet were given to each of them. It should be noted that all ethical principles, norms and international standards concerning the Helsinki declaration and the Convention on Human Rights and Biomedicine will be followed, respected, and preserved [24].

The evaluations were performed at the respective clubs’ facilities, where body composition and the vertical jump were performed in a room, and the RAST test was performed on the field. Groups of three players were evaluated each time, and the instruments were always in the same place to allow all players to have the same conditions to perform them. As mentioned above, the data collection was done with groups of three players each time, and during the tests, at least two people present specialized in performing them to maximize time and provide better knowledge of the tests in question.

### 2.5. Statistical Analysis

#### 2.5.1. Preliminary Analysis

An inspection of the data revealed no missing values, nor were univariate outliers found. A priori power analysis through G*Power [25] was used to determine the required sample size considering the following input parameters: effect size f = 0.45; *α* = 0.05; statistical power = 0.95. The required sample size was 81 (27 for each group), which was respected in the present study.

#### 2.5.2. Main statistical Analysis

Data were analyzed using the Statistical Package for the Social Sciences (SPSS) v. 23.0 (IBM, Chicago, IL, USA). Descriptive statistics were used to calculate means, standard deviation, minimum and maximum values. To check the normality of data distribution, the Shapiro–Wilk test was used (*p* > 0.05—normal distribution). In this way, for the variables with a non-normal distribution, we used the non-parametric Kruskal-Wallis test, intending to verify if there were differences between the three groups under study. Once differences were found, post hoc multiple comparisons were performed to compare the groups’ results, two by two. ANOVA was used for the remaining variables with normal distribution. For these tests, the significance level was set to *α* < 0.05. Subsequently, an effect size analysis (Cohen d) was used to determine its magnitude, and the following cut-off values were considered: 0–0.2, trivial; 0.21–0.6, small; 0.61–1.2, moderate, 1.21–2.0, large; and >2.0, very large [26].

## 3. Results

**Figure 2:**
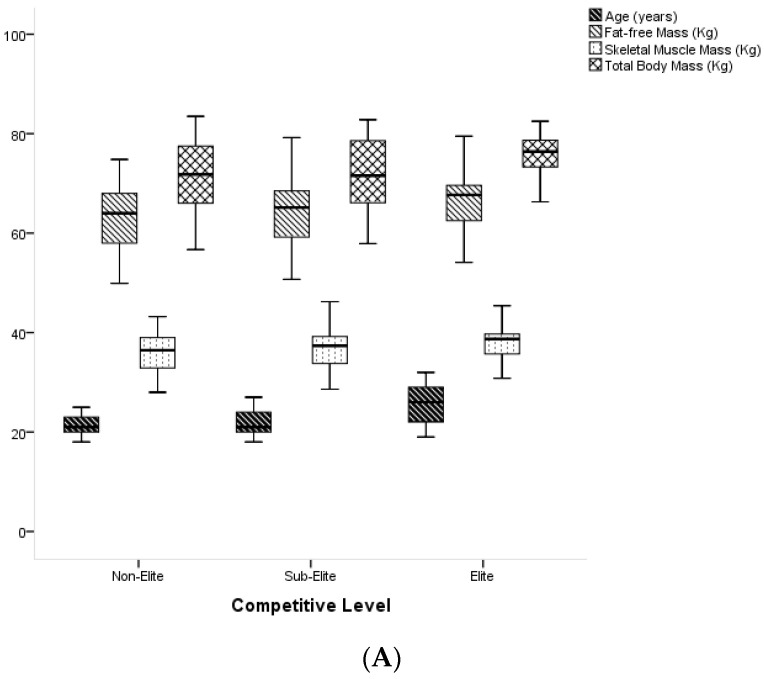

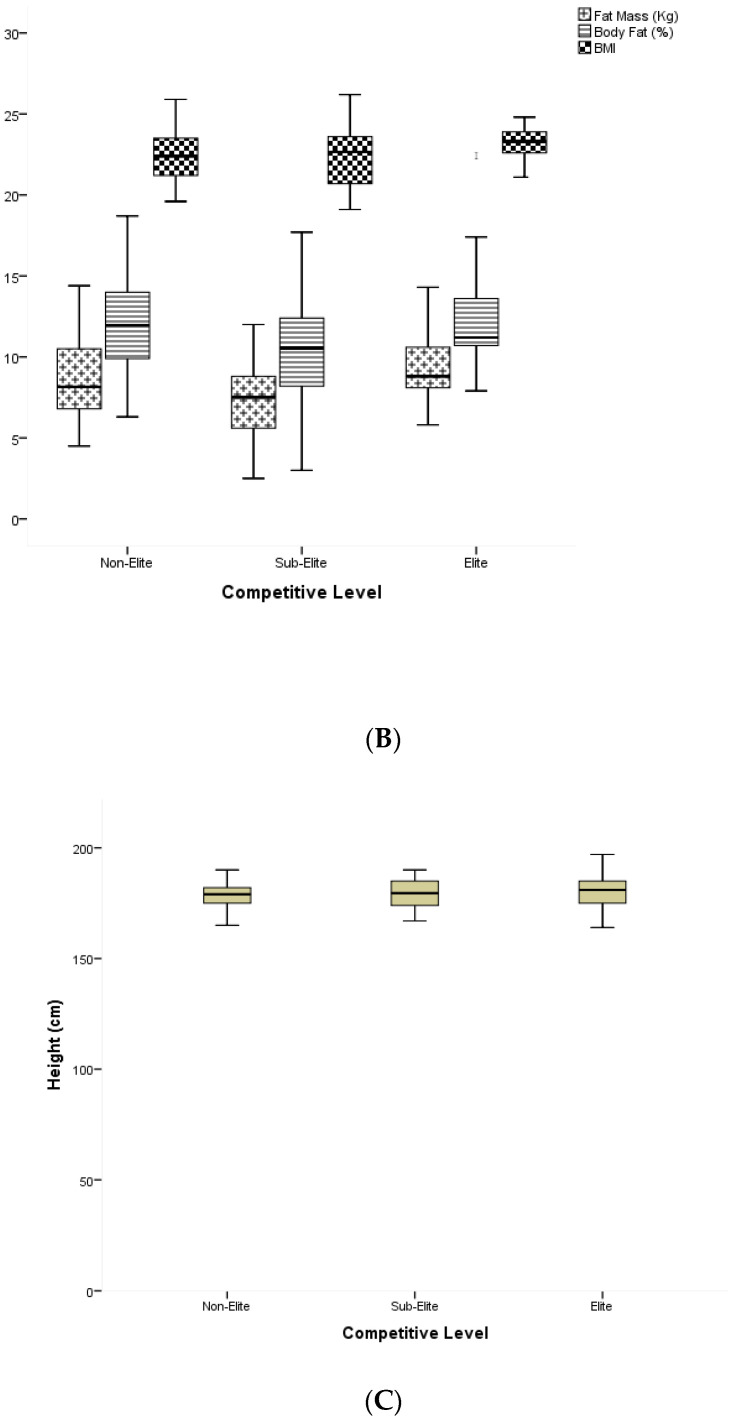
(**A**)—Comparisons between groups (competitive level) regarding the variables Age, Fat-free Mass, Skeletal Muscle Mass and Total Body Mass; (**B**)—Comparisons between groups, regarding the variables Fat Mass, Body Fat and BMI; (**C**)—Comparisons between groups, regarding the variable Height.

Figure 2 presents the data concerning the comparisons between the three groups (competitive level) regarding the variable age and body composition variables. There are statistically significant differences regarding the variable Age, comparing the Elite players with the Sub-Elite players (*p* = 0.001; Effect Size = 1.06), and comparing the Elite players with the Non-Elite players (*p* = 0.006; Effect Size = 0.69), with the Elite players having the highest mean age (25.67 ± 3.95).

Comparisons between the three groups (competitive level) regarding body composition variables showed statistically significant differences only for the variable Fat Mass, comparing Elite and Sub-Elite players (*p* = 0.04; Effect Size = 0.69), with Elite players having the highest average Fat Mass (9.5 ± 2.84).

Regarding the performance variables (lower limbs power—vertical jump), it is possible to analyze in Table 1 the comparisons between the three groups (competitive level). It can be verified that there are statistically significant differences for the variable Highest Jump, comparing the players of the three groups. For this variable, there were differences when comparing Elite players with Sub-Elite players (*p* = 0.022; Effect Size = 0.71) and between Non-Elite players and Sub-Elite players (*p* = 0.012; Effect Size = 0.76), with Sub-Elite players having the lowest mean values.

In Maximal Produced Strength, there are significant differences between Non-Elite and Sub-Elite players (*p* = 0.05; Effect Size = 0.64) and between Elite and Sub-Elite players (*p* = 0.002; Effect Size = 1.00).

Finally, in Table 2, it can be verified that there are statistically significant differences regarding the variables Average Power, Maximum Power, and Minimum Power in the comparisons between the three groups regarding the anaerobic power performance variables. In the mean power evaluation, there are significant differences between Elite players and Non-Elite players (*p* < 0.001; Effect Size = 2.43) and between Elite players and Sub-Elite players (*p* < 0.001; Effect Size = 2.22), obtaining the Elite players the highest/favorable mean values, comparing to the other two competitive levels. For maximum power, significant differences were found between Elite players and Non-Elite players (*p* < 0.001; Effect Size = 2.16) and between Elite players and Sub-Elite players (*p* < 0.001; Effect Size = 2.06). Finally, regarding the minimum power, we find differences between Elite players and Non-Elite players (*p* < 0.001; Effect Size = 2.16) and between Elite players and Sub-Elite players (*p* < 0.001; Effect Size = 1.95) where again Elite players got the highest/favorable mean values, comparing to the other two competitive levels.

## 4. Discussion

This study aimed to evaluate the body composition, lower limbs power, and anaerobic power of soccer players according to their competitive level. When consulting studies related to the addressed subject, no investigations were found comparing the three mentioned variables in three distinct competitive levels. However, there are several studies where these variables are evaluated using the same methods.

Regarding the age of the players, the present study shows that there are significant differences when comparing the three competitive levels, where the Elite players have a higher age. Also, the study of Haugen, Tønnessen, and Seiler [27] found similar results. In the present study, this result reflects that, possibly, Elite players are older due to the difficulty of reaching these competitive levels, probably being necessary to go through other competitive levels and obtain a more significant competitive experience until they can reach this professional level.

Regarding body composition components, no significant differences were found in most variables except for the variable Fat Mass, between the Elite and Sub-Elite players, predicting that these characteristics are similar in players, regardless of the competitive level in which they participate. This fact is corroborated by Santos [28] and Nobre et al. [29], which also refer to this homogeneity. On the other hand, Haugen et al. [27] present significant differences between the body composition of players from different competitive levels, showing that players from higher competitive levels present better results, a fact also verified in the present study. These shared characteristics may mean that, despite the differences between the demands of each competitive level, the players present identical characteristics between themselves. Given the typical demands required by the modality and possibly, independently of the competitive level of each player, these potentiate high caloric expenditure and eventually will be careful with their nutrition because an energetic consumption and a balanced alimentary diet form a better way to maintain a healthy and adequate body composition profile [30].

Another objective of the present study was to evaluate the players’ performance in the execution of the vertical jump; in this case, we found significant differences in the comparison between the three competitive levels, where the Sub-Elite players presented the worst results. Some studies refer that no significant differences were found, as was the case of the studies of Santos [28], Ribeiro, Dias, Claudino, and Gonçalves, [31], and the study done by Haugen et al. [27], the performance of the vertical jump is not able, by itself, to discriminate players of different competitive levels. In this way, in the present study, Non-Elite and Elite players present similar vertical jump values. Eventually, one of the possible reasons for these results may be due to the fact that, in the Elite players, besides the fact that some of these characteristics are also present, such as the physical disputes to get the ball, it can also be due to the higher demand of the competitive level, both in training and in the game. The maximum power produced by a player in the vertical jump showed significant differences between Non-Elite and Sub-Elite players and Elite and Sub-Elite players. This way, it is possible to refer that the Elite players present higher strength and power indexes than the other groups. This characteristic can also demonstrate that the physical demand is even higher than the Non-Elite players.

The performance of the players presented in evaluating the anaerobic power showed significant differences between the three competitive levels evaluated, where it can be verified that the Elite players obtained better values of Maximum Power, Minimum Power, and Average Power in comparison with the other levels. Additionally, the studies of Spigolon, Borin, Leite, Padovani and Padovani, [32], Pellegrinotti et al. [33], and Moro et al. [34] show similar results, the first two comparing different categories (juniors and seniors). On the other hand, the studies of Ribeiro et al. [31] and Alves et al. [35], also developed with junior players, do not indicate significant differences between the groups evaluated. In the present study, one can explain this occurrence with the fact that Elite players may present a better performance and can produce greater amounts of force and be more powerful, given the greater physiological demands that this professional level requires. There are also more training units to which they are subjected, something that may favor the development of this ability; thus, the performance of anaerobic power improves as the competitive level increases [34].

Regarding IF, the values found show a good capacity of the players to react to effort regardless of their competitive level. However, no significant differences were found between the three competitive levels. In the present study, this result can be explained by the fact that the evaluations were done in the middle of the season, and the players already had good physical preparation. In turn, Alves et al. [35] refer that players with lower values of lean mass are usually less resistant to these efforts, something that is observed when we compare these variables in junior players.

Following the methodology applied and the results obtained, some of the limitations found are mentioned, followed by suggestions for future research. One of the study’s limitations is related to the effort management of the players, where given the periodization established by each of the technical teams, the participation of all the players became impossible. Another limitation was related to the region/area of the country where the research was carried out, the interior region (Castelo Branco and Covilhã), which affects the number of teams that participated. Also, interference with the specific work of each team in their weekly microcycle conditioned the time available to carry out the evaluations and the availability of each team to collect data. All teams asked that the minimum time possible was taken up to not interfere with their training.

Future studies may choose to use the evaluation of these variables in soccer players; it is suggested that correlations be made between the performance of players considering their body composition, the inclusion of aerobic capacity, lactate and Squat Jump measurements, tactical and cognitive/decision-making variables. The tests for the individual evaluations could be a valuable tool to understand other existing differences. Finally, it could be useful to evaluate athletes in the training of different competitive levels and compare them with senior athletes and verify if the maturation conditions these variables.

## 5. Conclusions

The results found through this study indicate that Elite players are older. However, no significant differences were found in any body composition components, except for variable Fat Mass, this showed statistically significant differences only for the comparing Elite and Sub-Elite players, so it can be highlighted that there is homogeneity between the players relative to the body composition profile. In turn, in the performance tests, it was possible to verify that the Elite players present better values in all the variables than the lower competitive levels, so it can be considered that these players present better physical/athletic characteristics. Therefore, the physical/motor performance of the players can be a discriminatory criterion of the competitive level.

In summary, the study brings relevant information in the sense that the dissemination of the results/conclusions obtained provide information to coaches, technicians, and even players in order to, in practice, know the different characteristics of the players, depending on the competitive level. It is also essential to highlight the importance of evaluating these variables for a better knowledge of the players and as a training control tool.

## Figures and Tables

**Table 1:** Comparisons between groups (competitive level) regarding the athletes’ performance variables in the CMJ test—Lower limbs power/vertical jump.

Dependent Variable	Competitive Level	*n*	Mean ± SD	Effect Size (d; ±95% CI)	Sig.
Highest Jump (cm)	Non-Elite	30	37.66 ± 4.78	0.76 (0.23 ± 1.28) Moderate	0.012 *
	Sub-Elite	30	32.66 ± 7.89		
	Non-Elite	30	37.66 ± 4.78	0.02 (−0.54 ± −0.57) Trivial	0.999
	Elite	21	37.74 ± 5.55		
	Sub-Elite	30	32.66 ± 7.89	0.71 (0.14 ± 1.29) Moderate	0.022 *
	Elite	21	37.74 ± 5.55		
Maximum Power Produced (Watts)	Non-Elite	30	956.12 ± 103.40	0.64 (0.12 ± 1.16) Moderate	0.050
	Sub-Elite	30	882.83 ± 122.02		
	Non-Elite	30	956.12 ± 103.40	0.43 (−0.13 ± 1.00) Small	0.344
	Elite	21	1003.69 ± 115.11		
	Sub-Elite	30	882.83 ± 122.02	1.00 (0.41 ± 1.59) Moderate	0.002 *
	Elite	21	1003.69 ± 115.11		

* *p* < 0.05—significance level.

**Table 2:** Comparisons between groups (competitive level) regarding the athletes’ performance in Anaerobic power’s RAST test.

Dependent Variable	Competitive Level	*n*	Mean ± SD	Effect Size (d; ±95% CI)	Sig.
Average Power (Watts/Kg)	Non-Elite	30	9.76 ± 1.32	0.03 (−0.47 ± 0.54) Trivial	0.990
	Sub-Elite	30	9.81 ± 1.54		
	Non-Elite	30	9.76 ± 1.32	2.43 (1.70 ± 3.17) Very Large	<0.001 *
	Elite	21	13.46 ± 1.72		
	Sub-Elite	30	9.81 ± 1.54	2.22 (1.52 ± 2.93) Very Large	<0.001 *
	Elite	21	13.46 ± 1.72		
Maximum Power (Watts/Kg)	Non-Elite	30	11.09 ± 1.39	0.06 (−0.45 ± 0.56) Trivial	0.985
	Sub-Elite	30	11.17 ± 1.46		
	Non-Elite	30	11.09 ± 1.39	2.16 (1.46 ± 2.85) Very Large	<0.001 *
	Elite	21	14.59 ± 1.86		
	Sub-Elite	30	11.17 ± 1.46	2.06 (1.37 ± 2.75) Very Large	<0.001 *
	Elite	21	14.59 ± 1.86		
Minimum Power (Watts/Kg)	Non-Elite	30	8.68 ± 1.44	0.06 (−0.45 ± 0.57) Trivial	0.974
	Sub-Elite	30	8.58 ± 1.85		
	Non-Elite	30	8.68 ± 1.44	2.16 (1.46 ± 2.86) Very Large	<0.001 *
	Elite	21	12.29 ± 1.90		
	Sub-Elite	30	8.58 ± 1.85	1.98 (1.28 ± 2.63) Large	<0.001 *
	Elite	21	12.29 ± 1.90		
Fatigue Index (Watts/Sec)	Non-Elite	30	5.64 ± 2.48	0.16 (−0.35 ± 0.67) Trivial	0.828
	Sub-Elite	30	6.11 ± 3.29		
	Non-Elite	30	5.64 ± 2.48	0.28 (−0.28 ± 0.84) Small	0.650
	Elite	21	6.42 ± 3.04		
	Sub-Elite	30	6.11 ± 3.29	0.10 (−0.46 ± 0.65) Trivial	0.933
	Elite	21	6.42 ± 3.04		

* *p* < 0.05—significance level.
